# Mercury accumulator plants with phytoremediation potential in a region of northwestern Colombia

**DOI:** 10.1007/s11356-025-37078-9

**Published:** 2025-11-04

**Authors:** Lina Mosquera Chaverra, Diego Paredes Cuervo, Ana López Gutiérrez

**Affiliations:** 1 Faculty of Engineering, Environmental Engineering, Universidad Tecnológica del Choco Diego Luis Cordoba, Quibdó, 270002 Colombia; 2 Faculty of Environmental Sciences, Universidad Tecnológica de Pereira, Pereira, 660003 Colombia; 3 Faculty of Agricultural Sciences and Agroindustry, Universidad Tecnológica de Pereira, Pereira, 660003 Colombia

**Keywords:** Colombia, Floristic composition, Gold mining, Mercury, Plants, Phytoremediation

## Abstract

Mercury (Hg) is a toxic and nonessential metal for living organisms, and its environmental contamination poses a global concern. Gold mining is one of the primary sectors responsible for mercury emissions, which contributes significantly to ecosystem pollution. In this study, the natural regeneration of vegetation was evaluated at seven mining sites with varying periods of inactivity, and the accumulation of mercury in plant species was analyzed. Random plots were established at each site, where species were identified and selected to determine mercury concentrations in their tissues and calculate bioconcentration (BCF), translocation (TF), and accumulation (AF) factors. A total of 2505 individuals were identified from 46 species and 20 families, with a predominance of herbaceous species. The most widely distributed species, found at more than four study sites, included *Homolepis aturensis*, *Andropogon bicornis*, *Clidemia capitellata*, *Tonina fluviatilis*, *Pityrogramma calomelanos*, *Cespedesia spathulata*, and *Fuirena robusta*. The total mercury (THg) concentrations varied, ranging from 29.0 to ~ 21,500 ng g^−1^ in soils, < 20.0 to ~ 11,000 ng g^−1^ in roots, and < 20.0 to ~ 6500 ng g^−1^ in shoots. The BCF, TF, and AF values ranged from 0.14 to 1.96, 0.16 to 5.3, and 0.12 to 4.45, respectively. However, TF values may lack precision, as mercury in shoots could result from both translocation from roots and atmospheric deposition. *Miconia reducens*, *Cecropia peltata*, and *Pityrogramma calomelanos* presented the highest BCF values, indicating their potential for phytoremediation of mercury-contaminated soils in areas affected by gold mining.

**Supplementary Information:**

The online version contains supplementary material available at 10.1007/s11356-025-37078-9.

## Introduction

Mercury (Hg) is a highly toxic heavy metal, and its complex chemodynamics makes it a persistent environmental pollutant (Marrugo-Negrete et al. 2015). It is released from both natural sources and anthropogenic activities, causing severe ecological damage and posing serious risks to human health. One of its most worrying effects is its capacity to accumulate and biomagnify through trophic levels, disrupting food chains and leading to carcinogenic, teratogenic, neurological, and renal disorders, which can be fatal in severe cases (Xia et al. 2010; Mahbub et al. 2017).

Chemical speciation of mercury is varied, including metallic or elemental mercury (Hg^0^), inorganic mercury (Hg^+^ and Hg^2+^), and organic mercury, such as methylmercury (MeHg) and dimethylmercury (Me_2_Hg) (Priyadarshanee et al. 2022). Among these, methylmercury is the most toxic as a result of its high bioaccumulation capacity. In aquatic ecosystems, elemental mercury is converted to methylmercury by bacteria, facilitating its entry and accumulation in aquatic organisms and further propagation through ecosystems. Furthermore, its volatility and persistence in the environment allow for long-range transport, making it a global pollutant with severe implications for all living organisms (Calabrese et al. 2018).

According to the United Nations Environment Program (UNEP), global mercury emissions in 2015 reached 2220 tons, with artisanal and small-scale gold mining (ASGM) being the most significant contributor, accounting for 38% of total emissions, particularly in South America and Sub-Saharan Africa (UNEP – United Nations Environment Programme 2002). For example, in ASGM processes, mercury is used for gold amalgamation; however, improper handling of mercury has led to contamination of various environmental compartments (Marrugo-Negrete et al. 2016). Given this issue, remediation of mercury-contaminated sites, especially in mining areas, has become an urgent but challenging task (Xun et al. 2017). Although traditional remediation technologies such as chemical precipitation, extraction, thermal treatment, encapsulation, and electrodialysis are available, they are costly, labor-intensive, and generate hazardous waste, complicating their disposal (Mahbub et al. 2017; Raj and Maiti 2019).

In this context, more sustainable technologies, such as phytoremediation, have emerged as promising alternatives. Phytoremediation is a biological remediation technology that employs plants and their associated rhizospheric microorganisms to remove or immobilize heavy metals from the environment (Singh et al. 2023). The ideal plant species for this purpose must tolerate high metal concentrations, develop deep root systems, produce high biomass, and exhibit rapid growth rates (van der Ent et al. 2013; Xun et al. 2017). Mercury translocation from roots to shoots is not a universal trait among plants; it primarily occurs in certain species known as phytoaccumulators. In these plants, significant removal of Hg from contaminated soils is possible due to their ability to translocate mercury to aboveground tissues. In contrast, other species contribute to Hg stabilization by immobilizing the metal within the root zone or soil matrix, thereby reducing its bioavailability and environmental risk (Teng et al. 2020). However, the efficiency of mercury accumulation or stabilization depends largely on its chemical form and bioavailability in the soil solution (Millán et al. 2006; Lu et al. 2016). Additionally, plants can absorb elemental mercury (Hg^0^) from the atmosphere through leaf stomata (Patra and Sharma 2000) or through foliar uptake via atmospheric deposition (Millhollen et al. 2006).

In recent years, the capacity of more than 200 plant species, mainly herbaceous and perennial, to accumulate mercury has been examined (Marrugo-Negrete et al. 2016; Xun et al. 2017; Qian et al. 2018; Durante-Yánez et al. 2022; Cui et al. 2023; de Freitas et al. 2024; dos Santos Soares et al. 2025). However, to date, no recognized hyperaccumulator of mercury has been identified, and the concentration threshold for hyperaccumulators remains uncertain. Some studies have proposed that hyperaccumulator species may accumulate over 10 mg kg^−1^ mercury (Lasat 2002; Marrugo-Negrete et al. 2015).

Importantly, the total concentrations of mercury (THg) in plants growing at Hg mining sites are significantly high (Qian et al. 2018). These plants exhibit greater accumulation capacities and tolerance to potentially harmful contaminants than do those growing in noncontaminated soils (Durante-Yánez et al. 2022). Consequently, the selection of locally tolerant plant species or the use of dominant plant species for phytoremediation has become a viable alternative approach to soil restoration (Xiao et al. 2018; Zhu et al. 2018).

In this study, we hypothesized that plant species growing in areas disturbed by mining activity and contaminated with mercury could be used in remediation processes. Accordingly, the objective of this work was to evaluate the diversity of plant species in post-mining areas, quantify mercury concentrations, and assess their potential for phytoremediation. To this end, mercury concentrations were determined in plant tissues and rhizosphere soils, and bioconcentration (BCF) and translocation factors (TF) were calculated to identify the most suitable species for ecological restoration. Although previous research in the department of Chocó has described the floristic composition and successional dynamics of vegetation in mining-disturbed areas, no studies to date have quantified mercury accumulation in these plants or evaluated their phytoremediation potential. This work, therefore, represents the first systematic assessment of Hg accumulation and translocation in naturally regenerating species from ASGM-impacted soils in the Chocó, providing critical insights for the selection of native species adapted to the region’s specific edaphoclimatic conditions for use in ecological restoration strategies.

## Materials and methods

### Study area

**Fig. 1:**
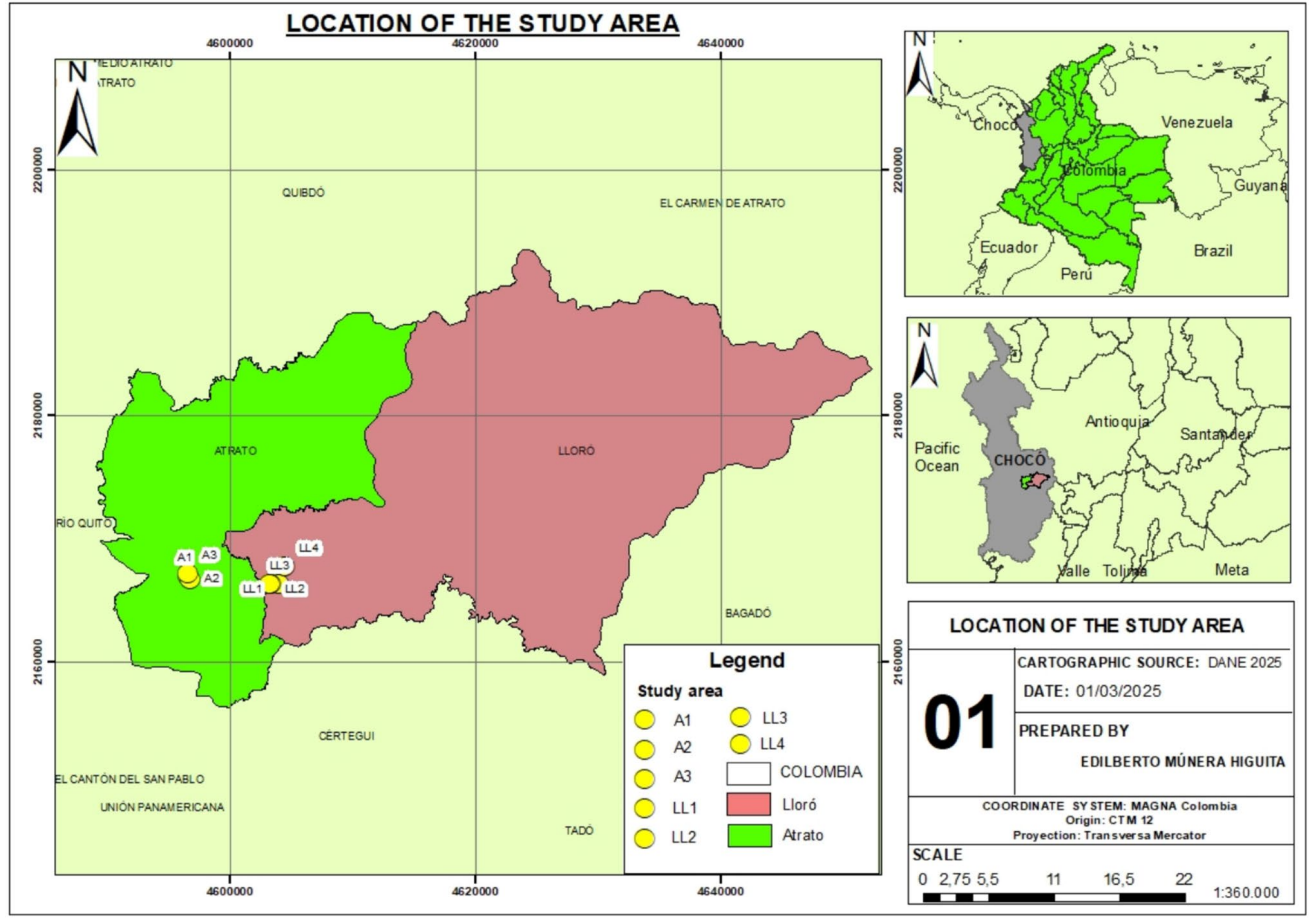
Location of the study area

This study was carried out in the municipalities of Atrato and Lloró, which are located in the Chocó department, one of the most biodiverse regions in western Colombia (Fig. 1). The area receives annual precipitation ranging from 5000 to 12,000 mm (Palomino-Ángel et al. 2019) and experiences minimum temperatures between 22 and 23 °C and maximum temperatures between 27 and 31 °C (Asprilla et al. 2018). These climatic conditions have contributed to the formation of ecologically significant ecosystems, such as tropical rainforests and wetlands (Palomino-Ángel et al. 2019; Córdoba-Tovar et al. 2023).

The study sites were selected because of the strong influence of gold mining, which is the main economic activity in the region. Within each municipality, the sampling areas included postmining sites with different abandonment periods, which means that they experienced no ongoing disturbance and were naturally colonized by plant species. The sampling campaigns were conducted in September 2023 and May 2024.

### Sample collection and pretreatment

Different sampling sites were selected in each locality on the basis of varying disturbance periods. Consequently, three and four study sites were chosen in the municipalities of Atrato and Lloró, respectively (Fig. 1). Within each site, linear transects measuring 2 m × 50 m were established to conduct a rapid flora biodiversity assessment. Three individuals of the same species of similar size were randomly selected from each sampling unit. A total of 297 plant samples were collected during the fieldwork. In addition, plant samples were collected for subsequent taxonomic identification. To complement the sampling strategy, three randomly selected individuals of the same species, along with their corresponding rhizosphere soil, were collected from a nearby area with no historical records of mining activity or mercury use. This sampling aimed to establish a reference for mercury concentrations in both soils and plant tissues under non-contaminated conditions.

Soil samples were collected directly beneath the canopy of the sampled plants. A composite topsoil sample (0–20 cm depth) was obtained by mixing three subsamples collected via plastic shovels (Marrugo-Negrete et al. 2016). For wetland species, plant samples were collected along the edges of water bodies. After collection, the plant samples were rinsed in the wetlands within the study sites. The samples were then separated into roots and aerial shoots via stainless steel scissors, placed in clean polyethylene plastic bags, and labeled accordingly with the corresponding soil samples. All samples were stored in ice-covered coolers until transportation to the laboratory.

Furthermore, two individuals of each plant species were pressed to establish the respective botanical herbarium samples, sprayed with a generous amount of alcohol for preservation, and later taxonomically identified at the Herbarium of the Universidad Tecnológica del Chocó Diego Luis Córdoba (CHOCO). Both terrestrial and amphibious plant species, including herbaceous, shrub, and tree growth forms, were considered.

At the Biological Processes Research Laboratory of the Universidad Tecnológica de Pereira (UTP) in Pereira, Colombia, plant samples were thoroughly washed with tap water, followed by three rinses with distilled water and a final rinse with deionized water to remove any adhering sediment particles. The separated plant tissues (roots and aerial shoots) were dried at 40 °C in a forced convection oven until they reached a constant weight. The dried plant tissues were then ground, placed in polyethylene bags, and stored at 4 °C in a cool environment until total mercury (THg) determination. Similarly, rhizosphere soil samples were air-dried at a constant weight, ground, and sieved before being stored in polyethylene bags at 4 °C until further analysis. All mercury determinations in plant tissues and soils were performed in triplicate (*n* = 3) to ensure analytical robustness.

Similarly, atmospheric mercury emissions in the air (Hg^0^) were determined via atomic absorption spectrometry with Zeeman background correction via a LUMEX RA-915 M device.

### Floristic composition and dominant plants

Within each defined transect (2 m × 50 m) at each sampling site, four random quadrants (each measuring 2 m × 12.5 m) were selected to assess the diversity of the plant species. In each quadrant, the total number of individuals was recorded, considering their growth habits during the sampling. The study sites were classified according to the time elapsed since mining activities ceased.

Several parameters, including relative density (RD), relative frequency (RF), and the importance value index (IVI), were calculated to characterize the vegetation structure. Additionally, biodiversity indices such as the Shannon–Wiener index (H’) and Simpson’s index (S) were determined. These parameters were calculated as follows:

1 $$\text{RD }\left(\text{\%}\right)= \frac{\text{Individual Plant }{\text{Species}}_{\text{number}} }{\text{Total number of individuals of all Plant species}}\times 100$$

2 $$\text{RF }\left(\text{\%}\right)= \frac{\text{Individual Plant }{\text{Species}}_{\text{frequency}} }{\text{Sum of frequencies of all species}} \times 100$$

3 $$\text{IVI }\left(\text{\%}\right)=\text{RA}+\text{RF}$$

4 $${H}{\prime}= -\sum Pi*lnPi$$

H: Shannon‒Wiener index; Pi = relative abundance; Ln: natural logarithm

5 $$S=1/\sum \frac{{n}_{i}({n}_{i}-1)}{N(N-1)}$$

*S*: Simpson index; *n*_*i*_: number of individuals of the *i*th species; *N*: total number of individuals.

### Determination of the total mercury concentration

The total mercury (THg) in the plant and soil samples was determined via a Zeeman RA-915 M mercury analyzer with the RAPID software (Lumex, Canada) in combination with a pyrolysis unit (PYRO-915 +). This method is based on thermal decomposition without prior sample pretreatment. THg quantification was performed via calibration curves constructed considering the area and mass of mercury (ng). The measurement of mercury absorption (peak height or peak area) is measured at 254 nm per mercury atom using the Zeeman correction for background absorption (Villamil 2015). Calibration curves were considered acceptable when the coefficient of determination (*R*^2^) was ≥ 0.99.

Approximately 200 mg of dried soil was weighed via an analytical balance with a precision of 0.1 mg. Similarly, for plant tissue analysis (shoots and roots), approximately 100 mg of dried material was used. All measurements were performed in triplicate for both soil and plant samples.

Quality assurance was evaluated in triplicate using certified reference materials for tomato leaves (CRM 1753a, 34 ng g^−1^) and sandy loam soil (VHG-DS-100G, 4.70 mg kg^−1^). Furthermore, a soil sample was spiked with different concentrations of mercury using a 1000 mg L^−1^ Hg stock solution (traceable to the National Institute of Standards and Technology, NIST) and used as a control for every 20 samples. All mercury concentrations were expressed on a dry weight (dw) basis. The detection limit was 20.0 ng g^−1^, with recovery rates of 99% for soil and 97% for plant tissue.

### Calculation of the bioconcentration, translocation, and accumulation factors

The bioconcentration factor (BCF), translocation factor (TF), and accumulation factor (AF) were used to assess mercury enrichment in plants (Eqs. 6, 7, and 8). The BCF was calculated as the ratio of the mercury concentration in the roots to the mercury concentration in the soil, while the TF was determined as the ratio of the mercury concentration in the shoots to that in the roots (Yoon et al. 2006). AF, sometimes referred to in the literature as the shoot bioconcentration factor (BCF_shoot_), was defined as the ratio of the mercury concentration in the shoots to that soil (Marrugo-Negrete et al. 2016). This index was included in addition to the conventional BCF (root/soil) and TF (shoot/root) to provide a more detailed assessment of Hg distribution within plants. Plants with both BCFs and TFs greater than 1.0 (BCFs and TFs > 1.0) are considered potential candidates for phytoextraction (Yoon et al. 2006; Marrugo-Negrete et al. 2016).

6 $$\text{BCF}=\frac{{C}_{\text{plant }\_\text{root}}}{{C}_{\text{soil}}}$$

7 $$\text{TF}=\frac{{C}_{\text{plant}\_\text{shoot}}}{{C}_{\text{plant}\_\text{root}}}$$

8 $$\text{AF}=\frac{{C}_{\text{plant}\_\text{shoot}}}{{C}_{\text{soil}}}$$

where:

*C*_plant_root_: metal concentration in the roots.
*C*_plant_shoot_: metal concentration in aerial shoots (stems and leaves).
*C*_plant_soil_: metal concentration in the soil.

### Data analysis

Data were summarized either as the median (minimum–maximum) or as mean ± standard deviation (SD), depending on the data distribution. Specifically, the mercury concentrations presented in Table are reported as medians due to their non-normal distribution. In contrast, the data in Figure 3 were log10-transformed to achieve normality and are expressed as mean ± SD.

Statistical analyses were performed using the RStudio (version 4.2.2), Microsoft Excel (version 16.91), and GraphPad Prism (version 10.0). Normality was assessed using the Shapiro–Wilk test for datasets with fewer than 50 samples and the Kolmogorov–Smirnov test for datasets with more than 50 samples. If the data did not follow a normal distribution, they were log10-transformed.

In addition, Pearson or Spearman correlation analyses were conducted, depending on whether the data for each study area followed a normal distribution, to evaluate the relationship between total mercury (THg) concentrations in soils and plant tissues (roots and shoots). A significance level of *p* < 0.05 was used for all analyses.

## Results and discussion

### Floristic composition

A total of 2505 plants were identified in seven gold mining areas with different abandonment periods, corresponding to 46 species, 39 genera, and 20 families. Among the total number of individuals recorded, 1370 were found in the municipality of Lloró and 1132 were found in Atrato. In general, an increase in family diversity was observed as the abandonment period increased. This pattern was reflected in study areas A1 (Atrato) and LL3 (Lloró), where 24 and 18 species were recorded, respectively (Table 1). However, at the individual level, the LL4 site in Lloró presented greater richness in terms of families, genera, and species compared to LL1 and LL2, which had longer abandonment periods.

**Table 1:** Distribution of the number of individuals identified in the study areas

Municipality	Study areas	Time of cessation of mining activity (years)	Families	Genera	Species	Number of individuals
Atrato	A1	5.0	16.0	22.0	24.0	465
	A2	3.0	10.0	14.0	15.0	237
	A3	1.5	7.0	13.0	14.0	430
Lloró	LL1	5.0	6.0	7.0	7.0	240
	LL2	3.0	5.0	6.0	6.0	271
	LL3	7.0	14.0	16.0	18.0	500
	LL4	0.33	8.0	13.0	15.0	362

Study areas: A1 (Atrato study area 1), A2 (Atrato study area 2), A3 (Atrato study area 3), Ll1 (Lloró study area 1), Ll2 (Lloró study area 2), Ll3 (Lloró study area 3), and Ll4 (Lloró study area 4)

**Fig. 2:**
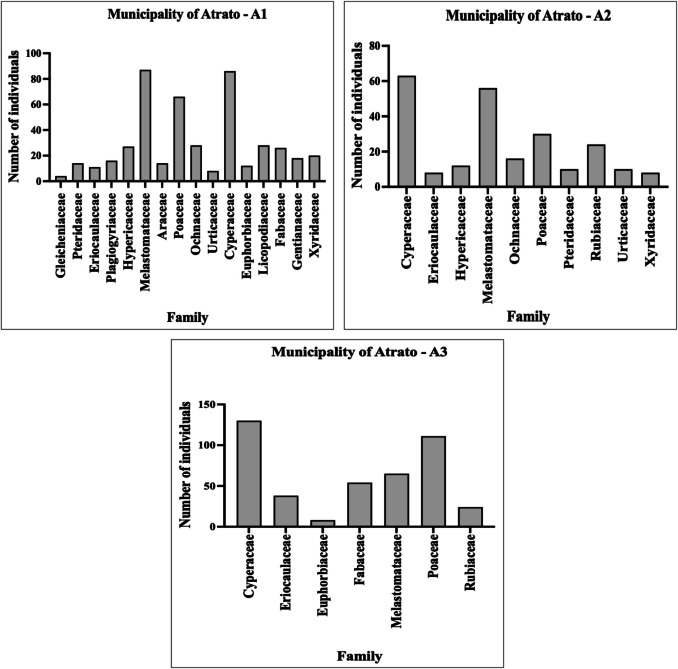

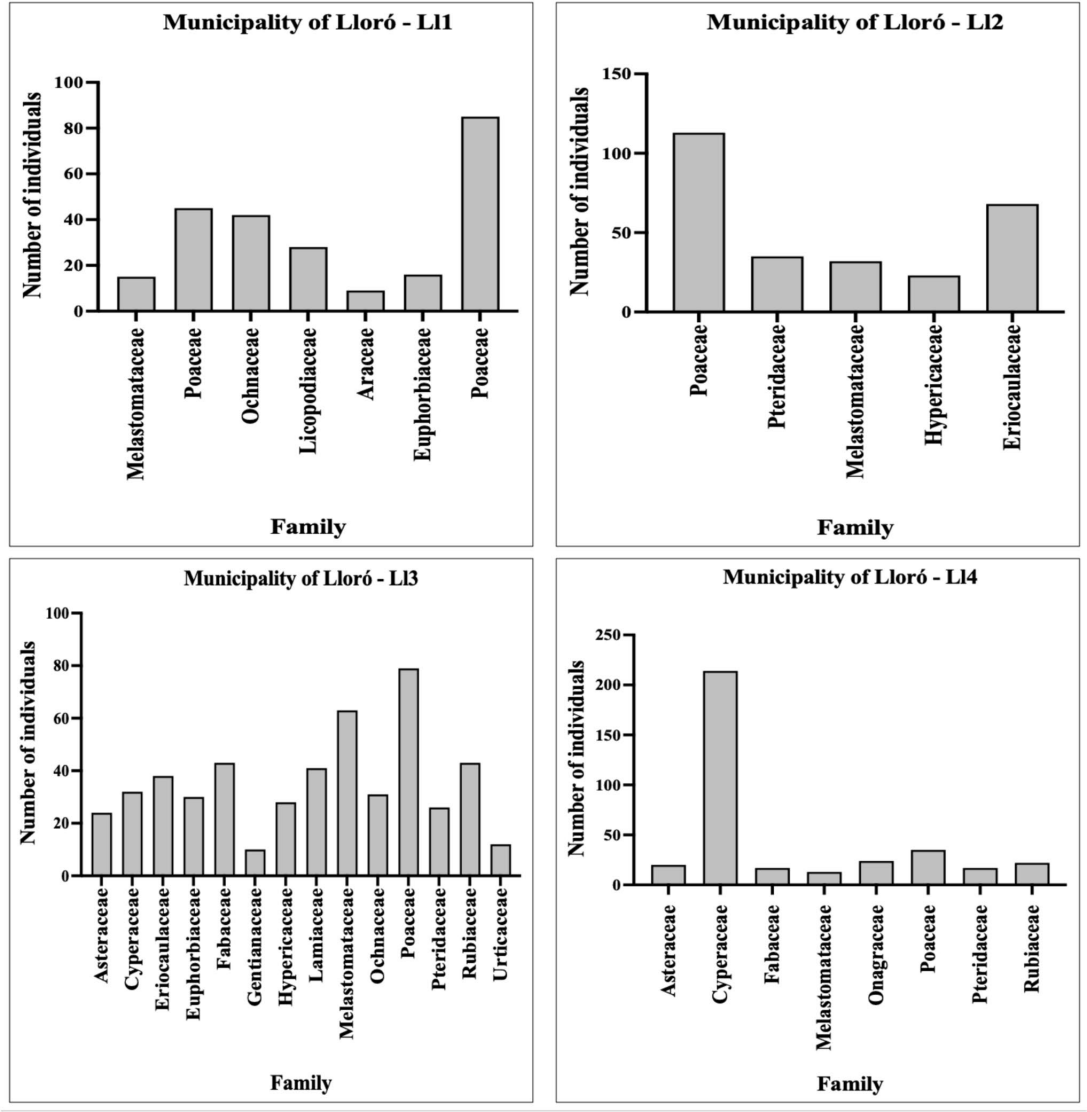
Taxonomic distribution of plant families recorded in the study areas. The *Y*-axis indicates the total number of individuals per family, grouped by study zone (Atrato: A1–A3; Lloró: Ll1–Ll4)

Among the study areas, the most representative families, considering the number of genera and species, were Cyperaceae, Melastomataceae, and Poaceae (Fig. 2; Table S1). Species from the Cyperaceae family are recognized as pioneer plants in terms of ecosystem colonization and formation (Gil et al. 2006; Marrugo-Negrete et al. 2016). The predominance of in mining-disturbed areas suggests that certain taxa within this family form functional groups capable of thriving under the harsh conditions of these habitats, progressively facilitating the recruitment of less tolerant species (Valois-Cuesta et al. 2022).

With respect to plant life forms (Table S1), the vegetation was predominantly composed of herbaceous species, which accounted for 72.72% of the total. This finding is consistent with those of previous studies (Marrugo-Negrete et al. 2016; Qian et al. 2018; Xiao et al. 2018; Zhu et al. 2018; Wu et al. 2021). In particular, owing to their small and lightweight seeds, herbaceous plants can easily disperse through (Li et al. 2007; Mikołajczak et al. 2017). In addition, these plants exhibit adaptive traits such as rapid propagation and germination, well-developed root systems, high growth rates, and the ability to establish themselves in barren sites, including those under environmental stress. For these reasons, herbaceous species are considered pioneer plants and are more likely to survive in contaminated areas (Yang et al. 2014; Marrugo-Negrete et al. 2016; Zhu et al. 2018).

### Dominant species and vegetation diversity

The floristic composition and dominant plant species in the different study areas are shown in Table 2 and Table S2. The Shannon‒Wiener and Simpson indices indicated greater diversity in A1, followed by Ll3 and A2. This suggests that the municipality of Atrato presented greater diversity of plant species than Lloró did, which is consistent with the data reported in Table 1.

**Table 2:** Characteristics of the species identified in more than four study areas

Study area	Number of individuals	%RD	%RF	Importance value index (IVI)	Simpson index	Shannon‒Wiener index	Species
Ll1	45	18.75	21.1	39.8	4.7	1.7	*Andropogon bicornis*
	42	17.5	15.8	33.3			*Cespedesia spathulata*
	85	35.4	21.1	56.5			*Homolepis aturensis*
Ll2	40	14.8	15	29.8	5.2	1.7	*Andropogon bicornis*
	73	26.9	20.0	46.9			*Homolepis aturensis*
	35	12.9	20.0	32.9			*Pityrogramma calomelanos*
	68	25.1	15	40.1			*Tonina fluviatilis*
Ll3	31	6.2	7.5	13.7	14.5	2.8	*Cespedesia spathulata*
	21	4.2	7.5	11.7			*Clidemia capitellata*
	79	15.8	7.5	23.3			*Homolepis aturensis*
	26	5.2	3.8	9			*Pityrogramma calomelanos*
	38	7.6	7.5	15.1			*Tonina fluviatilis*
Ll4	13	3.6	5.0	8.6	12.8	2.6	*Clidemia capitellata*
	25	6.9	10.0	16.9			*Fuirena robusta*
	17	4.7	5.0	9.7			*Pityrogramma calomelanos*
A1	28	6.0	6.3	12.3	19.3	3	*Cespedesia spathulata*
	32	6.9	4.8	11.7			*Clidemia capitellata*
	23	4.9	4.8	9.7			*Fuirena robusta*
	49	10.5	6.3	16.8			*Homolepis aturensis*
	14	3.0	3.2	6.2			*Pityrogramma calomelanos*
	11	2.4	4.8	7.2			*Tonina fluviatilis*
A2	12	5.1	9.5	14.6	14	2.6	*Andropogon bicornis*
	16	6.8	4.8	11.6			*Cespedesia spathulata*
	21	8.9	9.5	18.4			*Clidemia capitellata*
	14	5.9	4.8	10.7			*Fuirena robusta*
	10	4.2	4.8	9.0			*Pityrogramma calomelanos*
	8	3.4	4.8	8.2			*Tonina fluviatilis*
A3	38	8.8	9.3	18.1	11.2	2.5	*Andropogon bicornis*
	27	6.3	9.3	15.6			*Clidemia capitellata*
	33	7.7	9.3	17.0			*Fuirena robusta*
	73	17.0	9.3	26.3			*Homolepis aturensis*
	38	8.8	9.3	18.1			*Tonina fluviatilis*

The % RF (relative frequency), % RD (relative density), Simpson index, and Shannon‒Wiener index were calculated considering all species within each study zone

The families Poaceae (*Homolepis aturensis* and *Andropogon bicornis*), Melastomataceae (*Clidemia capitellata*), Eriocaulaceae (*Tonina fluviatilis*), Pteridaceae (*Pityrogramma calomelanos*), Ochnaceae (*Cespedesia spathulata*), and Cyperaceae (*Fuirena robusta*) dominated the vegetation structure of the study areas, as they were present in more than four of the seven study zones.

The study areas, analyzed individually, presented significant differences in plant communities according to the duration of disturbance. *Homolepis aturensis* was the species with the highest importance index (IVI) in zones Ll1, Ll2, Ll3, A1, and A3. On the other hand, in Ll4 and A2, it was *Fuirena robusta*. It is worth mentioning that these species were found in more than three delimited plots.

In general, the values of the ecological indices indicate that the study zones present significantly different levels of floristic diversity. According to Asquith (2002), the degree of disturbance in a forest plays a determining role in the recovery of its floristic diversity, often resulting in specific differences among secondary forests with varying degrees of succession**.**

In the early stages of succession (the first years after the cessation of disturbance), herbaceous species tend to dominate the vegetation. However, these species may be gradually replaced by plants with different life forms as the recovery of the ecosystem progresses. This phenomenon is evident in the present study, where species such as *Cecropia peltata*, *Vismia baccifera*, *Vismia macrophylla*, and *Cespedesia spathulata* dominated the arboreal stratum in zones with more advanced succession. These species not only stand out for their ecological role but also have great potential for reforestation programs in areas degraded by mining or abandoned lands (Valois-Cuesta and Martínez-Ruiz 2017).

### Mercury concentrations in soils

Mercury naturally occurs in soils, with a global average content of approximately 0.06 μg/g (Berrow and Reaves 1984). According to Kabata-Pendias (2000), the critical mercury levels in the soil range from 0.3 to 5.9 μg/g. Additionally, the United Nations Environment Program (UNEP – United Nations Environment Programme 2002) has established critical limits to prevent the ecological effects of Hg in organic soils, setting thresholds between 0.07 and 0.30 mg/kg.

**Fig. 3:**
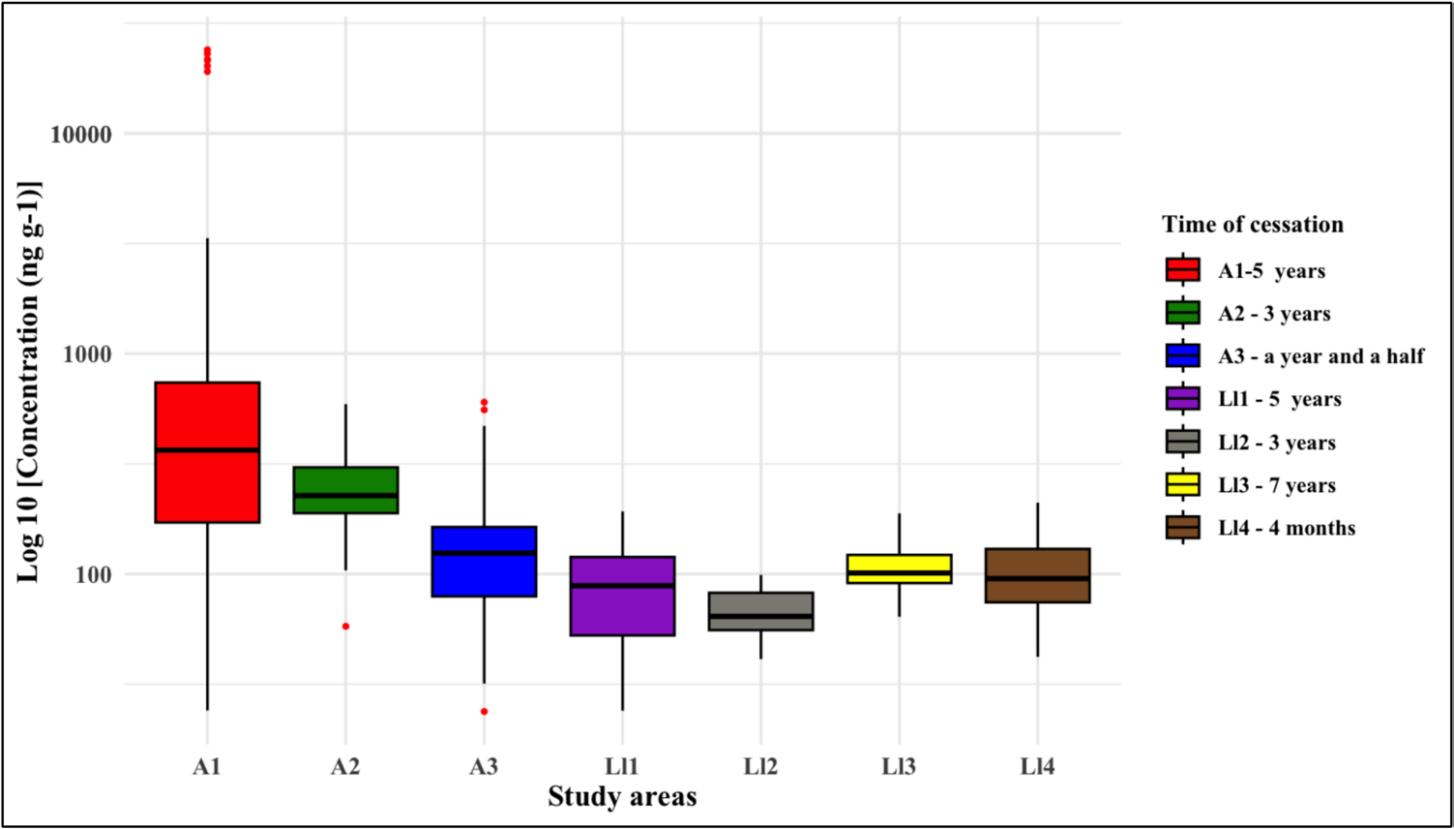
Box plot of log_10_-transformed total mercury (THg) concentrations (ng g^−1^) in soils across the study areas (Atrato: A1–A3; Lloró: Ll1–Ll4). Boxes indicate the interquartile range (IQR), horizontal lines within boxes represent the median, whiskers show the minimum and maximum values, and points denote outliers. Time since mining cessation for each site is shown in the legend

The concentrations of mercury in the soils in this study are presented in Fig. 3, with values ranging from 29.0 to 21,500 ng g^−1^. By study zone, the recorded concentrations were as follows: Ll1 (44.635–147.400 ng g^−1^), Ll2 (48.960–74.615 ng g^−1^), Ll3 (64.335–151.7 ng g^−1^), Ll4 (45.747–169.0 ng g^−1^), A1 (29.565–21,670 ng g^−1^), A2 (103.9–580.4 ng g^−1^), and A3 (52.590–556.0 ng g^−1^). In contrast, the rhizosphere soil collected from the reference site, an area with no known history of gold mining or mercury use, had THg concentrations below the method detection limit (< 20 ng g^−1^). This value confirms the very low natural background levels of mercury in the region and provides a baseline for interpreting contamination levels in mining-impacted areas.

According to Berrow and Reaves (1984), in many of the study areas, mercury concentrations can be considered as non-contaminated based on background levels reported in the literature. Typical background concentrations in soils range from 0.03 to 0.1 mg kg^−1^, whereas contaminated soils often present values that are two to four orders of magnitude higher (Li and Jia 2018; Wang et al. 2012). These findings support the interpretation that the elevated mercury levels observed in several sites in this study are primarily linked to anthropogenic inputs, particularly historical gold mining, rather than to natural background sources. Furthermore, Kot and Matyushkina (2002) indicate that soils with total mercury (THg) concentrations below 200 ng g^−1^ are classified as having low contamination; however, in zones where this threshold was exceeded, the potential use of these soils for agriculture or livestock production may pose a risk to public health.

When these results are compared with those of other studies, the mercury concentrations in this study are higher than those reported in the rural area of the municipality of Cláudia, Mato Grosso, Brazil (40.93–65.06 μg kg^−1^) (de Freitas et al. 2024). However, these concentrations are relatively low compared with those detected in the Almadén mining district in Spain (0.13–2695 μg g^−1^) (Molina et al. 2006), Mina Santa Cruz, Barranco Loba, Colombia (18.4 μg g^−1^) (Córdoba-Tovar et al. 2025), and the Wanshan district in China (0.74–1440 μg g^−1^) (Qian et al. 2018).

### Mercury accumulation in plants

The total concentrations of mercury (THg) in different plant tissues are presented in Table 3. In the roots, the values ranged from < 20.0 to 11,020.0 ng g^−1^, whereas in the shoots, the concentrations ranged from < 20.0 to 6913 ng g^−1^. In general, the plant species distributed across the study zones accumulated mercury in their tissues, indicating their ability to adapt to soils contaminated with this metal.

**Table 3:** Concentrations of total mercury (median standard ± deviation, ng g^−1^) in rhizosphere soils, roots, and shoots of the plant species from seven study areas, with associated bioconcentration (BCF), translocation (TF), and accumulation factors (AF)

Family	Scientific name	THg soil (ng g^−1^)	± SD	THg roots (ng g^−1^)	± SD	THg shoots (ng g^−1^)	± SD	BCF	TF	AF	Study areas
Araceae	*Syngonium podophillum*	804.500	202.712	639.500	324.040	694.000	134.584	0.795	1.085	0.863	A1
Cyperaceae	*Scleria secans*	404.750	31.183	95.935	3.147	159.050	47.588	0.237	1.658	0.393	
	*Cyperus luzulae*	115.210	41.847	61.635	12.622	71.280	12.162	0.535	1.156	0.619	
	*Eleocharis filiculmis*	112.850	11.809	72.405	2.369	43.420	6.392	0.642	0.600	0.385	
	*Fuirena robusta*	275.600	101.258	56.370	2.206	60.485	4.617	0.205	1.073	0.219	
Eriocaulaceae	*Tonina fluviatilis*	132.200	18.033	134.000	46.878	103.800	1.559	1.014	0.775	0.785	
Euphorbiaceae	*Phyllanthus caroliniensis*	253.850	14.779	79.240	0.042	68.345	15.408	0.312	0.863	0.269	
Fabaceae	*Mimosa pudica*	150.500	27.294	111.000	1.414	37.785	1.393	0.738	0.340	0.251	
Gentianaceae	*Chelonanthus alatus*	29.565	7.828	44.460	0.240	53.515	8.818	1.504	1.204	1.810	
Gleicheniaceae	*Sticherus bifidus*	2,017.000	103.504	1,082.000	53.818	1,500.000	25.541	0.536	1.386	0.744	
Hypericaceae	*Vismia macrophylla*	21,670.000	1,379.324	8,660.000	1,450.442	3,838.000	472.274	0.400	0.443	0.177	
	*Vismia baccifera*	609.650	237.941	119.400	6.081	159.150	6.859	0.196	1.333	0.261	
Licopodiaceae	*Palhinhaea cernua*	41.710	10.126	24.505	2.100	108.800	0.424	0.588	4.440	2.608	
Melastomataceae	*Clidemia hirta*	439.750	3.465	232.000	26.022	329.300	65.761	0.528	1.419	0.749	
	*Aciotis polystachya*	604.100	40.164	191.350	10.819	212.550	79.691	0.317	1.111	0.352	
	*Clidemia capitellata*	173.450	21.001	74.595	13.838	96.160	20.280	0.430	1.289	0.554	
	*Tibouchina herbacea*	233.700	19.092	100.510	4.087	228.200	21.779	0.430	2.270	0.976	
Ochnaceae	*Cespedesia spathulata*	392.800	185.545	362.000	155.563	278.550	65.549	0.922	0.769	0.709	
Plagiogyriaceae	*Plagiogyria euphlebia*	20,230.000	2,008.158	11,020.000	2,787.851	6,913.000	425.387	0.545	0.627	0.342	
Poaceae	*Pariana sp*	578.650	46.740	263.050	59.892	286.850	58.619	0.455	1.090	0.496	
	*Homolepis aturensis*	210.300	49.215	59.165	0.049	38.915	5.565	0.281	0.658	0.185	
Pteridaceae	*Pityrogramma calomelanos*	2,259.000	1,559.171	2,003.000	967.873	655.300	210.715	0.887	0.327	0.290	
Urticaceae	*Cecropia peltata*	473.750	99.914	362.000	180.595	401.950	63.145	0.764	1.110	0.848	
Xyridaceae	*Xyris jupicai*	267.200	36.487	153.050	15.486	56.315	0.856	0.573	0.368	0.211	
Cyperaceae	*Cyperus luzulae*	299.100	115.233	133.700	171.689	70.890	65.435	0.447	0.530	0.237	A2
	*Fuirena robusta*	222.900	73.429	181.100	140.986	156.800	104.139	0.812	0.866	0.703	
	*Eleocharis filiculmis*	111.600	7.637	156.200	51.195	52.645	11.858	1.400	0.337	0.472	
Eriocaulaceae	*Tonina fluviatilis*	186.250	4.313	111.350	1.909	67.205	6.512	0.598	0.604	0.361	
Hypericaceae	*Vismia baccifera*	219.150	10.394	175.300	0.566	61.135	5.890	0.800	0.349	0.279	
Melastomataceae	*Clidemia hirta*	222.300	179.626	207.900	93.175	334.300	93.981	0.935	1.608	1.504	
	*Miconia reducens*	243.950	62.155	479.900	234.618	844.050	146.018	1.967	1.759	3.460	
	*Clidemia capitellata*	580.400	13.576	305.000	30.971	143.600	32.951	0.525	0.471	0.247	
Ochnaceae	*Cespedesia spathulata*	188.600	27.003	211.400	82.540	204.900	402.790	1.121	0.969	1.086	
Poaceae	*Panicum polygonatum*	306.700	39.771	152.500	127.442	152.200	34.518	0.497	0.998	0.496	
	*Andropogon bicornis*	216.700	142.926	114.800	67.683	74.630	34.125	0.530	0.650	0.344	
Pteridaceae	*Pityrogramma calomelanos*	229.600	141.024	280.300	185.830	45.760	40.229	1.221	0.163	0.199	
Rubiaceae	*Psychotria poeppigiana*	226.450	13.506	285.100	116.955	646.450	37.547	1.259	2.267	2.855	
Urticaceae	*Cecropia peltata*	103.900	133.527	169.000	127.916	466.400	123.644	1.627	2.760	4.489	
Xyridaceae	*Xyris jupicai*	259.450	34.295	155.350	42.639	78.820	0.424	0.599	0.507	0.304	
Cyperaceae	*Rhynchospora tenerrima*	52.590	17.213	63.953	11.865	117.500	22.851	1.216	1.837	2.234	A3
	*Eleocharis filiculmis*	73.340	36.845	89.500	48.997	102.100	24.745	1.220	1.141	1.392	
	*Isolepis cernua*	85.410	44.449	152.143	60.984	82.91	28.599	1.781	0.545	0.971	
	*Scleria gaertneri*	556.000	67.141	94.013	3.732	68.46	6.182	0.169	0.728	0.123	
	*Fuirena robusta*	156.600	37.222	101.430	28.637	60.67	16.715	0.648	0.598	0.387	
Eriocaulaceae	*Tonina fluviatilis*	135.700	24.356	194.600	31.624	337.00	132.505	1.434	1.732	2.483	
	*Phyllanthus caroliniensis*	102.500	39.224	106.167	0.737	266.40	61.114	1.036	2.509	2.599	
Fabaceae	*Dalbergia monetaria*	150.700	34.427	197.233	6.451	111.50	53.463	1.309	0.565	0.740	
Melastomataceae	*Aciotis polystachya*	123.400	9.224	158.933	19.356	306.700	42.793	1.288	1.930	2.485	
	*Clidemia capitellata*	295.400	150.505	153.667	22.067	261.700	24.207	0.520	1.703	0.886	
	*Clidemia sericea*	179.500	49.178	166.400	42.316	258.80	53.026	0.927	1.555	1.442	
Poaceae	*Homolepis aturensis*	74.370	27.664	76.473	15.935	110.800	20.230	1.028	1.449	1.490	
	*Andropogon bicornis*	120.900	4.100	92.103	40.991	105.50	44.426	0.762	1.145	0.873	
Rubiaceae	*Psychotria poeppigiana*	314.300	56.322	217.200	33.207	334.00	29.509	0.691	1.538	1.063	
Melastomataceae	*Clidemia sericea*	44.815	18.042	61.881	6.053	51.338	8.973	1.381	0.830	1.146	Ll1
Araceae	*Epipremnum pinnatum*	87.500	16.360	117.433	11.380	86.04	15.565	1.342	0.733	0.983	
Euphorbiaceae	*Mallotus philippensis*	94.753	1.366	96.917	18.106	32.28	1.889	1.023	0.333	0.341	
Licopodiaceae	*Palhinhaea cernua*	147.400	19.337	83.063	16.257	87.78	32.118	0.564	1.057	0.596	
Ochnaceae	*Cespedesia spathulata*	132.207	62.963	169.133	14.263	73.360	20.876	1.279	0.434	0.555	
Poaceae	*Andropogon bicornis*	65.660	44.462	32.190	5.592	40.787	11.413	0.490	1.267	0.621	
	*Homolepis aturensis*	44.635	2.454	39.900	20.732	57.30	0.057	0.894	1.436	1.284	
Eriocaulaceae	*Tonina fluviatilis*	74.615	12.495	96.715	23.738	57.12	21.588	1.296	0.591	0.765	Ll2
Hypericaceae	*Vismia baccifera*	73.010	10.068	58.333	4.840	66.82	10.633	0.799	1.145	0.915	
Melastomataceae	*Tibouchina herbacea*	71.510	16.532	75.267	14.859	52.66	7.615	1.053	0.700	0.736	
Poaceae	*Andropogon bicornis*	62.680	24.858	20.163	0.427	16.80	6.312	0.322	0.833	0.268	
	*Homolepis aturensis*	73.025	15.563	10.288	1.459	29.85	4.271	0.141	2.901	0.409	
Pteridaceae	*Pityrogramma calomelanos*	48.960	7.567	64.880	10.224	27.65	3.467	1.325	0.426	0.565	
Pteridaceae	*Pityrogramma calomelanos*	127.250	13.930	89.055	6.965	103.870	34.266	0.700	1.166	0.816	LL3
Asteraceae	*Rolandra fruticosa*	78.830	0.438	108.185	27.598	90.875	5.933	1.372	0.840	1.153	
Cyperaceae	*Scleria secans*	114.700	3.677	60.500	0.453	27.770	1.626	0.527	0.459	0.242	
Eriocaulaceae	*Tonina fluviatilis*	93.297	18.230	51.540	16.150	36.880	14.631	0.552	0.716	0.395	
Euphorbiaceae	*Phyllanthus caroliniensis*	122.700	0.990	56.010	0.000	91.985	1.209	0.456	1.642	0.750	
Fabaceae	*Desmodium adscendens*	151.700	38.608	153.600	18.102	142.450	37.972	1.013	0.927	0.939	
	*Mimosa pudica*	128.000	21.213	42.885	5.395	90.260	15.330	0.335	2.105	0.705	
Gentianaceae	*Chelonanthus alatus*	94.170	0.537	59.680	0.255	96.440	2.319	0.634	1.616	1.024	
Hypericaceae	*Vismia baccifera*	64.335	0.559	84.145	14.729	51.870	4.978	1.308	0.616	0.806	
Lamiaceae	*Hyptis capitata J*	93.235	3.231	66.250	5.303	93.990	5.119	0.711	1.419	1.008	
Melastomataceae	*Clidemia capitellata*	84.985	1.068	41.790	14.284	67.530	10.805	0.492	1.616	0.795	
	*Clidemia hirta*	130.050	5.869	73.525	2.326	33.335	12.297	0.565	0.453	0.256	
	*Tibouchina herbacea*	99.930	11.978	56.035	1.450	196.800	0.566	0.561	3.512	1.969	
Ochnaceae	*Cespedesia spathulata*	107.850	5.586	60.255	0.813	35.065	5.466	0.559	0.582	0.325	
Poaceae	*Homolepis aturensis*	143.530	63.314	71.650	25.244	42.765	9.822	0.499	0.597	0.298	
Rubiaceae	*Spermacoce prostrata*	116.150	22.274	71.450	0.424	26.770	1.994	0.615	0.375	0.230	
	*Spermacoce alata*	79.510	8.796	28.300	3.917	32.635	0.389	0.356	1.153	0.410	
Urticaceae	*Cecropia peltata*	100.420	0.820	93.110	0.339	159.300	14.284	0.927	1.711	1.586	
Asteraceae	*Erechtites hieracifolia*	136.100	2.970	99.465	81.650	528.800	30.830	0.731	5.316	3.885	Ll4
Cyperaceae	*Diplacrum capitatum*	54.440	12.137	55.247	56.713	83.800	62.734	1.015	1.517	1.539	
	*Scleria gaertneri*	61.475	3.217	77.000	8.217	23.395	8.931	1.253	0.304	0.381	
	*Rhynchospora corymbosa*	88.607	0.618	58.933	18.933	42.490	9.400	0.665	0.721	0.480	
	*Eleocharis interstincta*	112.303	15.510	72.657	29.032	41.900	4.490	0.647	0.577	0.373	
	*Rhynchospora tenerrima*	169.000	58.831	138.850	23.547	135.900	32.527	0.822	0.979	0.804	
	*Scleria secans*	133.950	5.445	53.490	21.821	79.985	12.014	0.399	1.495	0.597	
	*Fuirena robusta*	142.300	4.243	100.080	27.464	137.250	6.718	0.703	1.371	0.965	
	*Cyperus odoratus*	108.500	5.940	119.250	1.061	132.950	5.020	1.099	1.115	1.225	
Fabaceae	*Mimosa pudica*	91.833	15.229	77.760	20.409	62.720	24.834	0.847	0.807	0.683	
Melastomataceae	*Clidemia capitellata*	45.747	4.038	47.103	23.772	40.970	30.898	1.030	0.870	0.896	
Onagraceae	*Ludwigia decurrens*	134.500	5.091	121.300	16.546	183.450	2.192	0.902	1.512	1.364	
Poaceae	*Panicum polygonatum*	118.450	15.768	41.810	16.532	33.070	3.677	0.353	0.791	0.279	
Pteridaceae	*Pityrogramma calomelanos*	80.545	10.246	101.250	1.768	101.600	27.436	1.257	1.003	1.261	
Rubiaceae	*Spermacoce alata*	88.035	10.260	74.400	34.068	101.525	27.825	0.845	1.365	1.153	

To establish a baseline for mercury accumulation in plants under non-contaminated conditions, three individuals of *Cespedesia spathulata* and *Cecropia peltata* were collected from a reference site, an area with no known history of gold mining or mercury use. Both species exhibited THg concentrations in roots and shoots below the method detection limit (< 20 ng g^−1^). These results confirm that, under non-contaminated conditions, mercury accumulation in plant tissues is negligible. The marked contrast between these baseline values and the elevated concentrations recorded in specimens from mining-impacted areas supports the conclusion that mercury uptake in the studied species is primarily driven by environmental contamination rather than by inherent physiological traits or natural background uptake. This evidence further emphasizes the importance of incorporating uncontaminated reference sites into bioaccumulation studies to effectively distinguish anthropogenic influences from natural variability.

For most species, roots accumulated higher mercury concentrations than shoots did, which agrees with the findings of previous studies (Marrugo-Negrete et al. 2016; de Freitas et al. 2024; dos Santos Soares et al. 2025). This phenomenon occurs because roots are in direct contact with mercury in the soil, where it primarily accumulates in the cell wall to minimize toxic effects on aerial parts, such as chlorosis and foliar necrosis (Marrugo-Negrete et al. 2015). Additionally, roots can immobilize heavy metals through adsorption or precipitation processes in the rhizosphere (Tangahu et al. 2011).

The highest concentrations of mercury in roots were found in Zone A1, suggesting greater availability of Hg in the soil in this area. The order of accumulation of mercury in roots across the study zones was A1 > A2 > A3 > Ll4 > Ll3 > Ll1 > Ll2, whereas in shoots, the observed pattern was A1 > A3 > A2 > Ll4 > Ll3 > Ll1 > Ll2. In our study, mercury concentrations in plant tissues were independent of the time elapsed since mining activities ceased. For example, at the time of sampling, A1 had been inactive for five years but still presented the highest mercury values in the roots and shoots (11,000 and 7,000 ng g^−1^, respectively). The five species with the highest concentrations of mercury in roots and shoots were found in the municipality of Atrato (A1), *P. euphlebia*, *V. macrophylla*, *P. calomelanos*, *S. bifidus*, and *S. podophillum* (Table 3).

Mercury in leaves can originate from both soil and atmospheric sources, as plants can absorb Hg in vapor form (Hg^0^) through stomatal and nonstomatal mechanisms (Azevedo and Rodriguez 2012; Marrugo-Negrete et al. 2016). Several studies have reported that a substantial proportion of Hg in leaf tissue can originate from atmospheric deposition, whereas root concentrations reflect soil mercury levels (Ericksen et al. 2003). Once accumulated in foliage, mercury can volatilize or return to the soil through leaf litter, contributing to its long-term persistence in vegetation and in soils (Liu et al. 2022).

Foliar Hg^0^ uptake is particularly relevant in areas with high mercury emissions, such as mining regions, smelters, or urban environments (Marrugo-Negrete et al. 2020; Casagrande et al. 2023). In this study, the temperatures exceeded 30 °C, which may have contributed to mercury volatilization, exacerbated by gold extraction activities that involve amalgam burning. In the municipality of Atrato, A1 recorded airborne mercury concentrations between 20 and 1800 ng m^−3^, exceeding the US EPA reference threshold (300 ng m^−3^) (US Epa 1995). In contrast, at other locations, the concentrations remained below 100 ng m^−3^, indicating that in some cases, airborne Hg concentrations may play a more significant role than soil Hg concentrations in plant metal accumulation.

A correlation analysis was performed in each of the seven study areas to assess the relationship between the concentration of THg in the soil and its accumulation in plant tissues (roots and shoots), as well as the correlation between these tissues (Table S3). In A1, a significant positive correlation was observed between the concentration of mercury in the soil and its accumulation in the 24 analyzed species: soil and roots (*r* = 0.860, *p* = 6.874e − 8), soil and shoots (*r* = 0.818, *p* = 9.99e − 07), and roots and shoots (*r* = 0.832, *p* = 4.612e − 07). In contrast, in A2, no significant correlation was detected between the soil mercury concentration and its accumulation in plant tissues or between roots and shoots in the 15 species evaluated (soil and roots:* r* = 0.260,* p* = 0.34; soil and shoots:* r* = 0.075, *p* = 0.790; roots and shoots: *r* = 0.467, *p* = 0.078). In A3, a significant positive correlation was found between roots and shoots, although the relationship between soil and plant tissues was not significant. With respect to the municipality of Lloró, a significant correlation was observed only in Ll4, both between the soil and roots (*r* = 0.532, *p* = 0.04) and between the soil and shoots (*r* = 0.589, *p* = 0.020), with an even stronger correlation between the roots and shoots (*r* = 0.703, *p* = 3.42e − 03).

In general, except for A1, the strongest correlations between the study zones were observed between the roots and shoots. A1 stood out as the area with the highest soil mercury concentrations (~ 20,000 ng g^−1^), suggesting that mercury accumulation in leaves may be more influenced by atmospheric Hg^0^ deposition than by soil absorption.

### Translocation, bioconcentration, and accumulation factors

In addition to evaluating the heavy metal content in plants, the bioconcentration factor (BCF) and the translocation factor (TF) provide more accurate representations of the metal accumulation and translocation characteristics in plants (Xiao et al. 2017). Therefore, studying these indices is essential to better understand the mechanisms of accumulation and transport in potentially metal-accumulating species (Wu et al. 2021). The BCF is a key index used to measure the ability of a plant to accumulate metals, whereas the TF reflects the efficiency with which total mercury (THg) is translocated from the roots to the aerial parts (Chang et al. 2020; Cui et al. 2023). Plants with high values of BCF and TF are more likely to extract metals from the soil and transport them to their aerial parts, making them potential candidates for phytoremediation processes (Xiao et al. 2018; Zhu et al. 2018). Table 3 presents the values of BCF, TF, and accumulation factor (AF) for the different analyzed species.

With respect to the bioconcentration factor (BCF), only 32% of the species studied presented values above 1.0, indicating an effective capacity for mercury accumulation. The species with the highest values in each study area were *Chelonanthus alatus* (1.50) in A1, *Miconia reducens* (1.96) in A2, *Isolepis cernua* (1.7) in A3, *Clidemia sericea* (1.38) in Ll1, *Pityrogramma calomelanos* in Ll2 and Ll4 (1.32 and 1.25, respectively), and *Rolandra fruticose* (1.37) in Ll3. These differences reflect the varying capacities for mercury uptake between species, which results from specific physiological strategies for metal absorption and tolerance (Rascio and Navari-Izzo 2011). Furthermore, BCF values are dependent not only on a plant’s ability to accumulate Hg but also on the bioavailability of the metal in the soil. In this study, the different cessation periods of mining activities in each area may have influenced Hg speciation, potentially leading to an underestimation of the actual capacity of the different plants to accumulate Hg.

For the translocation factor (TF), values above 1.0 indicate effective translocation of Hg from roots to shoots (Tu et al. 2002). This process can occur through evapotranspiration after root uptake (Tangahu et al. 2011; Manara 2012). Although most mercury is generally retained in the roots, some species develop specific mechanisms to facilitate its translocation (Shahid et al. 2017). This capability can increase tolerance to THg, which is predominantly absorbed by roots (da Silva et al. 2015). In our study, approximately 49% of the species presented TF values greater than 1.0. The species with the highest translocation capacity in each study area were *Palhinhaea cernua* (4.4) in A1, *Cecropia peltata* (2.7) in A2, *Phyllanthus caroliniensis* (2.5) in A3, *Homolepis aturensis* (1.4) in Ll1 and (2.9) in Ll2, *Tibouchina herbacea* (3.5) in Ll3, and *Erechtites hieracifolia* (5.3) in Ll4. A similar pattern has been reported in *Plectranthus* sp. (BCF: 0.33, TF: 1.73) and *Clidemia* sp. (BCF: 0.36, TF: 1.43), where THg concentrations were higher in leaves than in stems and roots (Marrugo-Negrete et al. 2016).

However, since these plants were collected from sites with different mining cessation periods, it was not possible to determine whether the mercury in the shoots originated exclusively from root translocation or was also absorbed directly from the atmosphere, as mercury can accumulate in aerial parts through foliar deposition. In areas with elevated atmospheric Hg^0^ emissions, such as artisanal and small-scale gold mining (ASGM) sites, shoots may incorporate mercury from both soil uptake and direct foliar absorption. This dual pathway can artificially increase TF values, creating the impression that a species has a greater translocation capacity than it possesses (Assad et al. 2016). In highly contaminated soils, less than 2% of total mercury is typically available for plant uptake (Dago et al. 2014), which further supports the possibility that a substantial fraction of Hg in aerial tissues may derive from atmospheric deposition. Consequently, some species with apparently high TF values may exhibit limited root-to-shoot mercury translocation. Given the specific environmental conditions in ASGM areas, where shots can accumulate mercury from multiple sources, the ideal phytoremediation species would be fast-growing, easy to harvest, and capable of storing high mercury concentrations in harvestable tissues. Future studies should incorporate experimental designs, such as isotopic tracing or controlled exposure, to better distinguish between mercury derived from roots and that deposited directly onto aerial plant parts.

Concerning the accumulation factor (AF), which reflects the proportion of metal in shoots relative to the soil along with harvestable biomass (Marrugo-Negrete et al. 2016), a value greater than 1.0 is considered efficient for foliar accumulation (de Freitas et al. 2024). In this study, 28% of the species presented AF values greater than 1.0. The species with the highest values in each study area were *Palhinhaea cernua* (2.6) in A1, *Cecropia peltata* (4.5) in A2, *Phyllanthus caroliniensis* (2.6) in A3, *Homolepis aturensis* (1.3) in Ll1, *Tibouchina herbacea* (1.9) in Ll3, and *Erechtites hieracifolia* (3.9) in Ll4. Importantly, when a plant exhibits good translocation or foliar absorption and primarily accumulates the metal in the shoots, harvesting the aerial parts can effectively remove the contaminant. In contrast, when metals accumulate mainly in the roots, remediation strategies may involve whole-plant extraction if the goal is contaminant removal. Alternatively, such species may serve as phytostabilizers, contributing to metal immobilization in the rhizosphere without the need for harvesting.

In summary, only 13 species presented consistent BCF, TF, and AF values above 1.0. In most cases, a high BCF value correlated with high TF and/or AF values. Since plants were collected from areas affected by small-scale and artisanal gold mining (ASGM), where shoots can accumulate mercury from both soil and air, THg absorption and translocation varied significantly between species and even within the same species depending on the study area. Furthermore, variations in accumulation patterns were observed among different genera and taxonomic families.

### Potential candidates for phytoremediation

Despite variations in mercury concentrations in soils at the seven study sites, most plant species demonstrated the ability to absorb. Herbaceous species accounted for 72% of the flora analyzed, and their high density and relative frequency indicate that these areas were rapidly colonized by species with this type of growth (Tables S1 and S2).

**Fig. 4:**
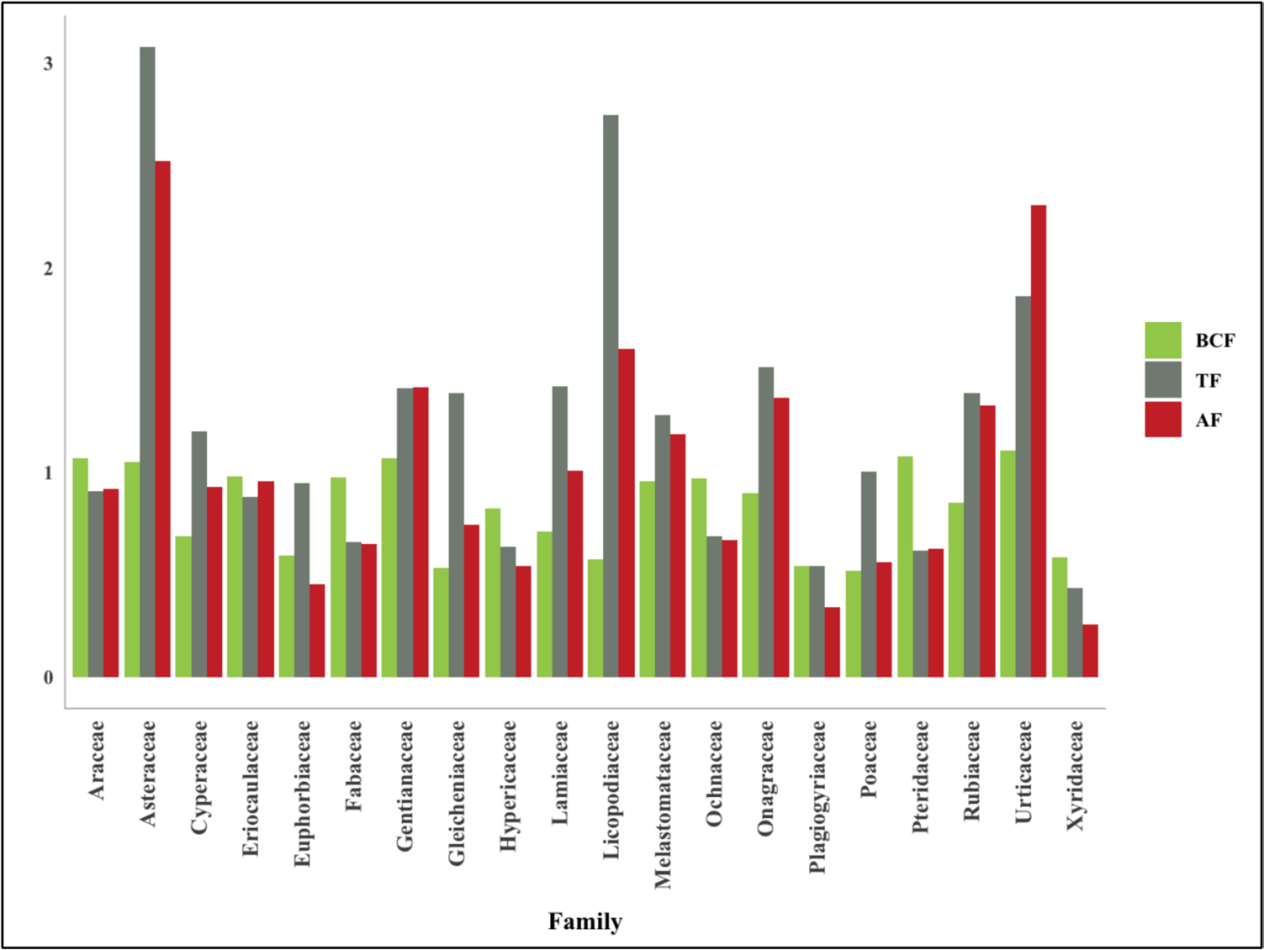
BCF, TF, and AF values in the identified families

The shrubs and subshrubs were represented by the families Euphorbiaceae, Hypericaceae, Melastomataceae, Rubiaceae, Fabaceae, Ochnaceae and Urticaceae. Among these, *Vismia macrophylla* grows in highly mercury-contaminated soils (~ 20,000 ng g^−1^) despite not having high BCF, TF, or AF values. Other tree species, such as *Cecropia peltata* and *Vismia baccifera*, presented BCF values above 0.7. Furthermore, the families Urticaceae and Asteraceae presented the highest values of BCF, TF, and AF, respectively (Fig. 4).

Given the critical role of soil in the bioavailability of mercury for plants and considering that BCF was the most relevant factor under the study conditions, species such as *Miconia reducens*, *Cecropia peltata*, and *Pityrogramma calomelanos* could be potential candidates for the phytoremediation of Hg-contaminated soil. In particular, *Cecropia peltata* has shown the ability to accumulate large amounts of Hg without exhibiting toxic effects (Vidal-Durango et al. 2010) and has been reported as an accumulator of Cd and Pb (Córdoba-Tovar et al. 2025), positioning it as a promising species for the restoration of degraded soil. Likewise, *Pityrogramma calomelanos* has been documented for Pb phytoremediation (Pulukkunadu Thekkeveedu and Hegde 2021).

Several of the species recorded in this study, including *Cecropia peltata*, *Cyperus luzulae*, *Eleocharis interstincta*, *Eleocharis filiculmis*, Pityrogramma calomelanos, *Mimosa pudica*, and *Erechtites hieracifolia*, have previously been reported in the literature as tolerant to mercury and capable of accumulating it in their tissues (de Freitas et al. 2024; dos Santos Soares et al. 2025; Marrugo-Negrete et al. 2016). This consistency with earlier findings strengthens the ecological relevance of the species identified in this study.

Although herbaceous species and subshrubs appear to be the most abundant and efficient phytoextractors due to their rapid growth, their lower biomass production could limit their remediation impact. In contrast, while trees accumulate less mercury, their high biomass production capacity may compensate for their relatively low metal accumulation efficiency, allowing them to remove significant amounts of contaminants. Additionally, trees do not need to be replanted every season and can live for decades or even centuries, which, together with occasional pruning, enables effective long-term remediation of contaminated sites. Therefore, its inclusion in ecological remediation strategies is essential (Marrugo-Negrete et al. 2016; Córdoba-Tovar et al. 2025).

Importantly, some of the species identified in this study have traditional uses among the inhabitants of Atrato and Lloró, primarily for medicinal and food purposes. However, given the ability of certain plants to translocate and accumulate Hg in their leaves, their consumption and use should be approached with caution to prevent mercury biomagnification in the food chain, which could pose risks to human health, livestock, and wildlife. This consideration is crucial when assessing their suitability for phytoremediation in mining-affected areas.

## Conclusions

This study demonstrates the variability in natural plant cover regeneration during the early stages of succession in mercury-contaminated areas, as a function of the time elapsed since the cessation of gold mining activities. A total of 2505 individuals belonging to 46 species and 20 families were recorded, with a clear predominance of herbaceous species. The most widely distributed species across the study areas included *Homolepis aturensis*, Andropogon *bicornis*, *Clidemia capitellata*, *Tonina fluviatilis*, *Pityrogramma calomelanos*, *Cespedesia spathulata*, and *Fuirena robusta*.

Analysis of mercury content in plant tissues revealed that several species are capable of tolerating and accumulating this metal, with varying efficiencies in its transfer from the soil to the roots (BCF) and from the roots to the shoots (TF). However, TF data were not conclusive due to the experimental conditions, and although they were calculated under the assumption that mercury in shoots originated exclusively from root-to-shoot translocation, in areas with high atmospheric Hg^0^ emissions, foliar uptake may also have contributed to Hg concentrations in aerial tissues. This dual accumulation pathway could lead to an overestimation of the actual translocation capacity.

Thirteen species exhibited BCF, TF, and AF values greater than 1.0, suggesting their potential for the phytoremediation of mercury-contaminated soils. Although *Vismia macrophylla* did not display high values for these indices, it was able to establish and grow in soils with elevated total mercury (THg) concentrations, indicating potential tolerance to contamination. In contrast, *Miconia reducens, Cecropia peltate*, and *Pityrogramma calomelanos* recorded the highest BCF values, standing out as species with greater potential for ecological remediation processes in tropical areas affected by mercury associated with gold mining.

To our knowledge, this is the first study in the department of Chocó that systematically quantifies Hg concentrations in these naturally regenerating species and evaluates their BCF and TF, providing novel evidence of their phytoremediation potential under ASGM conditions.

Future research should evaluate some of the species identified in this study under controlled conditions to precisely differentiate between mercury absorbed via roots and that deposited atmospherically, there by validating their true phytoremediation potential.

Overall, these findings provide relevant insights for the design of ecological restoration strategies in metal-contaminated soils and constitute a robust basis for the selection of species with potential applications in phytoremediation programs.

## Supplementary Information

Below is the link to the electronic supplementary material.

ESM 1 (DOCX 72.0 KB)

## Data Availability

All authors declare that all data and materials, and software application or custom code, support their published claims and comply with field standards.
