# Supplementary material for: Mercury accumulator plants with phytoremediation potential in a region of northwestern Colombia
Source: Environ Sci Pollut Res Int. 2025 Nov 4;32(44):25353–71. doi: 10.1007/s11356-025-37078-9 (PMC12647310; doi:10.1007/s11356-025-37078-9)
Supplement: Supplementary file 1 — (DOCX 72.0 KB) [file 11356_2025_37078_MOESM1_ESM.docx]

**Mercury accumulator plants with phytoremediation potential in a region of northwestern Colombia**

Lina Mosquera Chaverra^1^, Diego Paredes Cuervo^2*^, Ana López Gutiérrez^2^

^1^ Universidad Tecnológica del Chocó Diego Luis Cordoba, COLOMBIA

^2^ Universidad Tecnológica de Pereira, COLOMBIA

*Corresponding author. E-mail: [diparede@utp.edu.co](mailto:diparede@utp.edu.co)

**Table S1. Characteristics of plant species identified in the municipalities of Atrato and Lloró after the cessation of gold mining activities.**

| No | Family | Gender | Species | Growth form | Life form | Habit | Voucher Number | Municipality (Study areas) | | | | | | |
| --- | --- | --- | --- | --- | --- | --- | --- | --- | --- | --- | --- | --- | --- | --- |
|  |  |  |  |  |  |  |  | **Lloró - Ll1** | **Lloró - Ll2** | **Lloró - Ll3** | **Lloró - Ll4** | **Atrato - A1** | **Atrato - A2** | **Atrato - A3** |
| 1 | Araceae | *Epipremnum* | *Epipremnum pinnatum* | Herbaceous | Perennial | Epiphyte |  | X |  |  |  |  |  |  |
| 2 | Araceae | *Syngonium* | *Syngonium podophillum* | Herbaceous | Annual | Epiphyte | 00258 |  |  |  |  | X |  |  |
| 3 | Asteraceae | *Erechtites* | *Erechtites hieracifolia* | Herbaceous | Perennial | Terrestrial | 05077 |  |  |  | X |  |  |  |
| 4 | Asteraceae | *Rolandra* | *Rolandra fruticosa* Kuntze | Herbaceous | Perennial | Terrestrial | 01712 |  |  | X |  |  |  |  |
| 5 | Cyperaceae | *Cyperus* | *Cyperus luzulae* (L.) Rottb. ex Retz. | Herbaceous | Perennial | Amphibian | 04684 |  |  |  |  | X | X |  |
| 6 | Cyperaceae | *Cyperus* | *Cyperus odoratus* L. | Herbaceous | Annual | Amphibian | 01614 |  |  |  | X |  |  |  |
| 7 | Cyperaceae | *Diplacrum* | *Diplacrum capitatum* Boeckeler | Herbaceous | Perennial | Terrestrial | 03622 |  |  |  | X |  |  |  |
| 8 | Cyperaceae | *Eleocharis* | *Eleocharis filiculmis* Kunth | Herbaceous | Perennial | Amphibian | 02481 |  |  |  |  | X | X | X |
| 9 | Cyperaceae | *Eleocharis* | *Eleocharis interstincta* | Herbaceous | Perennial | Amphibian | 18140 |  |  |  | X |  |  |  |
| 10 | Cyperaceae | *Fuirena* | *Fuirena robusta* Kunth | Herbaceous | Perennial | Amphibian |  |  |  |  | X | X | X | X |
| 11 | Cyperaceae | *Isolepis* | *Isolepis cernua.* | Herbaceous | Annual | Amphibian |  |  |  |  |  |  |  | X |
| 12 | Cyperaceae | *Rhynchospora* | *Rhynchospora corymbosa* (L.) Britton | Herbaceous | Perennial | Amphibian | 01705 |  |  |  | X |  |  |  |
| 13 | Cyperaceae | *Rhynchospora* | *Rhynchospora tenerrima* Nees ex Spreng | Herbaceous | Perennial | Amphibian | 03015 |  |  |  | X |  |  | X |
| 14 | Cyperaceae | *Scleria* | *Scleria gaertneri* | Herbaceous | Perennial | Terrestrial | 14815 |  |  |  | X |  |  | X |
| 15 | Cyperaceae | *Scleria* | *Scleria secans* Urb. | Herbaceous | Perennial | Epiphyte | 00365 |  |  | X | X | X |  |  |
| 16 | Eriocaulaceae | *Tonina* | *Tonina fluviatilis* Aubl. | Herbaceous | Perennial | Amphibian | 18162 |  | X | X |  | X | X | X |
| 17 | Euphorbiaceae | *Mallotus* | *Mallotus philippensis* | Arboreal | Annual | Terrestrial |  | X |  |  |  |  |  |  |
| 18 | Euphorbiaceae | *Phyllanthus* | *Phyllanthus caroliniensis*Walter | Herbaceous | Perennial | Terrestrial | 29690 |  |  | X |  | X |  | X |
| 19 | Fabaceae | *Dalbergia* | *Dalbergia monetaria* L.F. | Shrub | Annual | Amphibian | 01436 |  |  |  |  |  |  | X |
| 20 | Fabaceae | *Desmodium* | *Desmodium adscendens* (Sw.) DC | Herbaceous | Perennial | Amphibian | 04355 |  |  | X |  |  |  |  |
| 21 | Fabaceae | *Mimosa* | *Mimosa pudica* L. | Herbaceous | Annual o Perennial | Terrestrial | 01487 |  |  | X | X | X |  |  |
| 22 | Gentianaceae | *Chelonanthus* | *Chelonanthus alatus* | Herbaceous | Annual | Terrestrial | 10045 |  |  | X |  | X |  |  |
| 23 | Gleicheniaceae | *Sticherus* | *Sticherus bifidus* (Willd.) Ching | Herbaceous | Annual | Terrestrial | 01494 |  |  |  |  | X |  |  |
| 24 | Hypericaceae | *Vismia* | *Vismia baccifera* Planch. & Triana | Arboreal | Annual | Terrestrial | 18204 |  | X | X |  | X | X |  |
| 25 | Hypericaceae | *Vismia* | *Vismia macrophylla* Kunth | Arboreal | Annual | Terrestrial | 09234 |  |  |  |  | X |  |  |
| 26 | Lamiaceae | *Hyptis* | *Hyptis capitata* Jacq | Herbaceous | Annual | Terrestrial | 00278 |  |  | X |  |  |  |  |
| 27 | Licopodiaceae | *Palhinhaea* | *Palhinhaea cernua* (L) | Herbaceous | Perennial | Terrestrial | 01827 | X |  |  |  | X |  |  |
| 28 | Melastomataceae | *Aciotis* | *Aciotis polystachya* (Bonpl) Triana | Herbaceous | Annual | Terrestrial | 15642 |  |  |  |  | X |  | X |
| 29 | Melastomataceae | *Clidemia* | *Clidemia capitellata* (Bonpl.) D. Don | Shrub | Perennial | Terrestrial | 10388 |  |  | X | X | X | X | X |
| 30 | Melastomataceae | *Clidemia* | *Clidemia hirta* (L) D. Don | Shrub | Perennial | Terrestrial | 13454 |  |  | X |  | X | X |  |
| 31 | Melastomataceae | *Clidemia* | *Clidemia sericea* | Shrub | Annual | Terrestrial |  | X |  |  |  |  |  | X |
| 32 | Melastomataceae | *Miconia* | *Miconia reducens* Triana | Shrub | Perennial | Terrestrial | 10330 |  |  |  |  |  | X |  |
| 33 | Melastomataceae | *Tibouchina* | *Tibouchina herbacea* | Shrub | Perennial | Terrestrial | 16802 |  | X | X |  | X |  |  |
| 34 | Ochnaceae | *Cespedesia* | *Cespedesia spathulata* | Arboreal | Perennial | Terrestrial | 13450 | X |  | X |  | X | X |  |
| 35 | Onagraceae | *Ludwigia* | *Ludwigia decurrens* Walter | Herbaceous | Perennial | Amphibian | 03244 |  |  |  | X |  |  |  |
| 36 | Plagiogyriaceae | *Plagiogyria* | *Plagiogyria euphlebia.* | Herbaceous | Perennial | Terrestrial |  |  |  |  |  | X |  |  |
| 37 | Poaceae | *Andropogon* | *Andropogon bicornis* L. | Herbaceous | Perennial | Amphibian | 15006 | X | X |  |  |  | X | X |
| 38 | Poaceae | *Homolepis* | *Homolepis aturensis* Chase | Herbaceous | Perennial | Terrestrial | 18154 | X | X | X |  | X |  | X |
| 39 | Poaceae | *Panicum* | *Panicum polygonatum* Schrad. | Herbaceous | Perennial | Amphibian | 54030 |  |  |  | X |  | X |  |
| 40 | Poaceae | *Pariana* | *Pariana* sp | Herbaceous | Perennial | Terrestrial | 77550 |  |  |  |  | X |  |  |
| 41 | Pteridaceae | *Pityrogramma* | *Pityrogramma calomelanos* Link | Herbaceous | Perennial | Terrestrial | 13197 |  | X | X | X | X | X |  |
| 42 | Rubiaceae | *Psychotria* | *Psychotria poeppigiana* Müll. Arg. | Shrub | Annual | Terrestrial | 14106 |  |  |  |  |  | X | X |
| 43 | Rubiaceae | *Spermacoce* | *Spermacoce alata* | Herbaceous | Perennial | Terrestrial |  |  |  | X | X |  |  |  |
| 44 | Rubiaceae | *Spermacoce* | *Spermacoce prostrata* | Herbaceous | Annual | Terrestrial |  |  |  | X |  |  |  |  |
| 45 | Urticaceae | *Cecropia* | *Cecropia peltata* L. | Arboreal | Perennial | Terrestrial | 11734 |  |  | X | X | X |  |  |
| 46 | Xyridaceae | *Xyris* | *Xyris jupicai* Rich. | Herbaceous | Perennial | Terrestrial | 03065 |  |  |  |  | X | X |  |

**Table S2. Ecological indices of plant species identified in the municipalities of Atrato and Lloró after the cessation of mining activity.**

| No | Specie | Time of cessation of activity (year) | Number of individuals | RD | %RD | RF | %RF | Importance Value Index (IVI) | Simpson Index | Shannon-Wiener index | Study Area |
| --- | --- | --- | --- | --- | --- | --- | --- | --- | --- | --- | --- |
| 1 | *Clidemia sericea* | 5.0 | 15 | 0.063 | 6.3 | 0.50 | 10.5 | 16.8 | 4.7 | 1.7 | Lloró - Ll1 |
| 2 | *Andropogon bicornis* L. |  | 45 | 0.188 | 18.8 | 1.00 | 21.1 | 39.8 |  |  |  |
| 3 | *Cespedesia spathulata* |  | 42 | 0.175 | 17.5 | 0.75 | 15.8 | 33.3 |  |  |  |
| 4 | *Palhinhaea cernua* (L) |  | 28 | 0.117 | 11.7 | 0.50 | 10.5 | 22.2 |  |  |  |
| 5 | *Epipremnum pinnatum* |  | 9 | 0.038 | 3.8 | 0.75 | 15.8 | 19.5 |  |  |  |
| 6 | *Mallotus philippensis* |  | 16 | 0.067 | 6.7 | 0.25 | 5.3 | 11.9 |  |  |  |
| 7 | *Homolepis aturensis* Chase |  | 85 | 0.354 | 35.4 | 1.00 | 21.1 | 56.5 |  |  |  |
| Total | |  | 240 | 1.000 | 100.0 | 4.75 | 100.0 | 200.0 |  |  |  |
| 1 | *Andropogon bicornis* L. | 3.0 | 40 | 0.148 | 14.8 | 0.75 | 15.0 | 29.8 | 5.2 | 1.7 | Lloró - Ll2 |
| 2 | *Pityrogramma calomelanos* Link |  | 35 | 0.129 | 12.9 | 1.00 | 20.0 | 32.9 |  |  |  |
| 3 | *Tibouchina herbacea* |  | 32 | 0.118 | 11.8 | 1.00 | 20.0 | 31.8 |  |  |  |
| 4 | *Vismia baccifera* Planch. & Triana |  | 23 | 0.085 | 8.5 | 0.50 | 10.0 | 18.5 |  |  |  |
| 5 | *Homolepis aturensis* Chase |  | 73 | 0.269 | 26.9 | 1.00 | 20.0 | 46.9 |  |  |  |
| 6 | *Tonina fluviatilis* Aubl. |  | 68 | 0.251 | 25.1 | 0.75 | 15.0 | 40.1 |  |  |  |
| Total | |  | 271 | 1.000 | 100.0 | 5.00 | 100.0 | 200.0 |  |  |  |
| 1 | *Pityrogramma calomelanos* Link | 7.0 | 26 | 0.052 | 5.2 | 0.5 | 3.8 | 9.0 | 14.5 | 2.8 | Lloró - Ll3 |
| 2 | *Desmodium adscendens* (Sw.) DC |  | 23 | 0.046 | 4.6 | 0.5 | 3.8 | 8.4 |  |  |  |
| 3 | *Rolandra fruticosa* Kuntze |  | 24 | 0.048 | 4.8 | 0.8 | 5.7 | 10.5 |  |  |  |
| 4 | *Mimosa pudica* L. |  | 20 | 0.040 | 4.0 | 0.5 | 3.8 | 7.8 |  |  |  |
| 5 | *Spermacoce prostrata* |  | 21 | 0.042 | 4.2 | 0.8 | 5.7 | 9.9 |  |  |  |
| 6 | *Clidemia capitellata* (Bonpl.) D. Don |  | 21 | 0.042 | 4.2 | 1.0 | 7.5 | 11.7 |  |  |  |
| 7 | *Clidemia hirta* (L.) D. Don |  | 24 | 0.048 | 4.8 | 0.5 | 3.8 | 8.6 |  |  |  |
| 8 | *Tonina fluviatilis* Aubl. |  | 38 | 0.076 | 7.6 | 1.0 | 7.5 | 15.1 |  |  |  |
| 9 | *Cecropia peltata* L. |  | 12 | 0.024 | 2.4 | 0.8 | 5.7 | 8.1 |  |  |  |
| 10 | *Homolepis aturensis* Chase |  | 79 | 0.158 | 15.8 | 1.0 | 7.5 | 23.3 |  |  |  |
| 11 | *Spermacoce alata* |  | 22 | 0.044 | 4.4 | 0.5 | 3.8 | 8.2 |  |  |  |
| 12 | *Chelonanthus alatus* |  | 10 | 0.020 | 2.0 | 0.3 | 1.9 | 3.9 |  |  |  |
| 13 | *Phyllanthus caroliniensis*Walter |  | 30 | 0.060 | 6.0 | 0.8 | 5.7 | 11.7 |  |  |  |
| 14 | *Vismia baccifera* Planch. & Triana |  | 28 | 0.056 | 5.6 | 1.0 | 7.5 | 13.1 |  |  |  |
| 15 | *Cespedesia spathulata* |  | 31 | 0.062 | 6.2 | 1.0 | 7.5 | 13.7 |  |  |  |
| 16 | *Hyptis capitata* Jacq |  | 41 | 0.082 | 8.2 | 0.8 | 5.7 | 13.9 |  |  |  |
| 17 | *Scleria secans* Urb. |  | 32 | 0.064 | 6.4 | 1.0 | 7.5 | 13.9 |  |  |  |
| 18 | *Tibouchina herbacea* |  | 18 | 0.036 | 3.6 | 0.8 | 5.7 | 9.3 |  |  |  |
|  | Total |  | 500 | 1.000 | 100.0 | 13.3 | 100.0 | 200.0 |  |  |  |
| 1 | *Clidemia capitellata* (Bonpl.) D. Don | 0.3 | 13.0 | 0.036 | 3.6 | 0.5 | 5.0 | 8.6 | 12.8 | 2.6 | Lloró - Ll4 |
| 2 | *Diplacrum capitatum* Boeckeler |  | 19.0 | 0.052 | 5.2 | 1.0 | 10.0 | 15.2 |  |  |  |
| 3 | *Pityrogramma calomelanos* Link |  | 17.0 | 0.047 | 4.7 | 0.5 | 5.0 | 9.7 |  |  |  |
| 4 | *Scleria gaertneri* |  | 10.0 | 0.028 | 2.8 | 0.3 | 2.5 | 5.3 |  |  |  |
| 5 | *Mimosa pudica* L. |  | 17.0 | 0.047 | 4.7 | 0.8 | 7.5 | 12.2 |  |  |  |
| 6 | *Rhynchospora corymbosa* (L.) Britton |  | 20.0 | 0.055 | 5.5 | 0.5 | 5.0 | 10.5 |  |  |  |
| 7 | *Panicum polygonatum* Schrad. |  | 35.0 | 0.097 | 9.7 | 0.5 | 5.0 | 14.7 |  |  |  |
| 8 | *Spermacoce alata* |  | 22.0 | 0.061 | 6.1 | 0.8 | 7.5 | 13.6 |  |  |  |
| 9 | *Eleocharis interstincta* |  | 53.0 | 0.146 | 14.6 | 0.3 | 2.5 | 17.1 |  |  |  |
| 10 | *Rhynchospora tenerrima* Nees ex Spreng |  | 27.0 | 0.075 | 7.5 | 0.8 | 7.5 | 15.0 |  |  |  |
| 11 | *Ludwigia decurrens* Walter |  | 24.0 | 0.066 | 6.6 | 1.0 | 10.0 | 16.6 |  |  |  |
| 12 | *Scleria secans* Urb. |  | 17.0 | 0.047 | 4.7 | 0.5 | 5.0 | 9.7 |  |  |  |
| 13 | *Erechtites hieracifolia* |  | 20.0 | 0.055 | 5.5 | 0.8 | 7.5 | 13.0 |  |  |  |
| 14 | *Fuirena robusta* Kunth |  | 25.0 | 0.069 | 6.9 | 1.0 | 10.0 | 16.9 |  |  |  |
| 15 | *Cyperus odoratus* L. |  | 43.0 | 0.119 | 11.9 | 1.0 | 10.0 | 21.9 |  |  |  |
|  | Total |  | 362 | 1.000 | 100 | 10 | 100 | 200 |  |  |  |
| 1 | *Sticherus bifidus* (Willd.) Ching | 5.0 | 4 | 0.009 | 0.9 | 0.3 | 1.6 | 2.4 | 19.3 | 3.0 | Atrato - A1 |
| 2 | *Pityrogramma calomelanos* Link |  | 14 | 0.030 | 3.0 | 0.5 | 3.2 | 6.2 |  |  |  |
| 3 | *Tonina fluviatilis* Aubl. |  | 11 | 0.024 | 2.4 | 0.8 | 4.8 | 7.1 |  |  |  |
| 4 | *Plagiogyria euphlebia*. |  | 16 | 0.034 | 3.4 | 0.8 | 4.8 | 8.2 |  |  |  |
| 5 | *Vismia macrophylla* Kunth |  | 17 | 0.037 | 3.7 | 0.8 | 4.8 | 8.4 |  |  |  |
| 6 | *Clidemia hirta* (L.) D. Don |  | 12 | 0.026 | 2.6 | 0.5 | 3.2 | 5.8 |  |  |  |
| 7 | *Syngonium podophillum* |  | 14 | 0.030 | 3.0 | 0.5 | 3.2 | 6.2 |  |  |  |
| 8 | *Pariana sp* |  | 17 | 0.037 | 3.7 | 0.5 | 3.2 | 6.8 |  |  |  |
| 9 | *Cespedesia spathulata* |  | 28 | 0.060 | 6.0 | 1.0 | 6.3 | 12.4 |  |  |  |
| 10 | *Cecropia peltata* L. |  | 8 | 0.017 | 1.7 | 0.5 | 3.2 | 4.9 |  |  |  |
| 11 | *Scleria secans* Urb. |  | 12 | 0.026 | 2.6 | 0.3 | 1.6 | 4.2 |  |  |  |
| 12 | *Aciotis polystachya* (Bonpl) Triana |  | 9 | 0.019 | 1.9 | 0.5 | 3.2 | 5.1 |  |  |  |
| 13 | *Phyllanthus caroliniensis* Walter |  | 12 | 0.026 | 2.6 | 0.5 | 3.2 | 5.8 |  |  |  |
| 14 | *Homolepis aturensis* Chase |  | 49 | 0.105 | 10.5 | 1.0 | 6.3 | 16.9 |  |  |  |
| 15 | *Clidemia capitellata* (Bonpl.) D. Don |  | 32 | 0.069 | 6.9 | 0.8 | 4.8 | 11.6 |  |  |  |
| 16 | *Cyperus luzulae* (L.) Rottb. ex Retz. |  | 15 | 0.032 | 3.2 | 0.8 | 4.8 | 8.0 |  |  |  |
| 17 | *Vismia baccifera* Planch. & Triana |  | 10 | 0.022 | 2.2 | 0.5 | 3.2 | 5.3 |  |  |  |
| 18 | *Tibouchina herbacea* |  | 34 | 0.073 | 7.3 | 1.0 | 6.3 | 13.7 |  |  |  |
| 19 | *Palhinhaea cernua* (L) |  | 28 | 0.060 | 6.0 | 0.8 | 4.8 | 10.8 |  |  |  |
| 20 | *Eleocharis filiculmis* Kunth |  | 36 | 0.077 | 7.7 | 1.0 | 6.3 | 14.1 |  |  |  |
| 21 | *Mimosa pudica* L. |  | 26 | 0.056 | 5.6 | 1.0 | 6.3 | 11.9 |  |  |  |
| 22 | *Fuirena robusta* Kunth |  | 23 | 0.049 | 4.9 | 0.8 | 4.8 | 9.7 |  |  |  |
| 23 | *Chelonanthus alatus* |  | 18 | 0.039 | 3.9 | 0.5 | 3.2 | 7.0 |  |  |  |
| 24 | *Xyris jupicai* Rich. |  | 20 | 0.043 | 4.3 | 0.5 | 3.2 | 7.5 |  |  |  |
|  | Total |  | 465 | 1.000 | 100 | 15.8 | 100.0 | 200.0 |  |  |  |
| 1 | *Cyperus luzulae* (L.) Rottb. ex Retz. | 3.0 | 24 | 0.101 | 10.1 | 0.8 | 7.1 | 17.3 | 14.0 | 2.6 | Atrato - A2 |
| 2 | *Pityrogramma calomelanos* Link |  | 10 | 0.042 | 4.2 | 0.5 | 4.8 | 9.0 |  |  |  |
| 3 | *Cecropia peltata* L. |  | 10 | 0.042 | 4.2 | 0.5 | 4.8 | 9.0 |  |  |  |
| 4 | *Clidemia hirta* (L.) D. Don |  | 17 | 0.072 | 7.2 | 0.8 | 7.1 | 14.3 |  |  |  |
| 5 | *Panicum polygonatum* Schrad. |  | 18 | 0.076 | 7.6 | 0.8 | 7.1 | 14.7 |  |  |  |
| 6 | *Andropogon bicornis* L. |  | 12 | 0.051 | 5.1 | 1.0 | 9.5 | 14.6 |  |  |  |
| 7 | *Cespedesia spathulata* |  | 16 | 0.068 | 6.8 | 0.5 | 4.8 | 11.5 |  |  |  |
| 8 | *Miconia reducens* Triana |  | 18 | 0.076 | 7.6 | 0.5 | 4.8 | 12.4 |  |  |  |
| 9 | *Fuirena robusta* Kunth |  | 14 | 0.059 | 5.9 | 0.5 | 4.8 | 10.7 |  |  |  |
| 10 | *Psychotria poeppigiana* Müll.Arg. |  | 24 | 0.101 | 10.1 | 1.0 | 9.5 | 19.7 |  |  |  |
| 11 | *Clidemia capitellata* (Bonpl.) D. Don |  | 21 | 0.089 | 8.9 | 1.0 | 9.5 | 18.4 |  |  |  |
| 12 | *Xyris jupicai* Rich. |  | 8 | 0.034 | 3.4 | 0.5 | 4.8 | 8.1 |  |  |  |
| 13 | *Vismia baccifera* Planch. & Triana |  | 12 | 0.051 | 5.1 | 0.8 | 7.1 | 12.2 |  |  |  |
| 14 | *Tonina fluviatilis* Aubl. |  | 8 | 0.034 | 3.4 | 0.5 | 4.8 | 8.1 |  |  |  |
| 15 | *Eleocharis filiculmis* Kunth |  | 25 | 0.105 | 10.5 | 1.0 | 9.5 | 20.1 |  |  |  |
|  | Total |  | 237 | 1.000 | 100 | 10.5 | 100.0 | 200.0 |  |  |  |
| 1 | *Rhynchospora tenerrima Nees ex Spreng* | 1.5 | 32 | 0.074 | 7.4 | 1.0 | 9.3 | 16.7 | 11.2 | 2.5 | Atrato - A3 |
| 2 | *Eleocharis filiculmis* Kunth |  | 28 | 0.065 | 6.5 | 0.5 | 4.7 | 11.2 |  |  |  |
| 3 | *Aciotis polystachya* (Bonpl) Triana |  | 16 | 0.037 | 3.7 | 0.8 | 7.0 | 10.7 |  |  |  |
| 4 | *Homolepis aturensis* Chase |  | 73 | 0.170 | 17.0 | 1.0 | 9.3 | 26.3 |  |  |  |
| 5 | *Clidemia capitellata* (Bonpl.) D. Don |  | 27 | 0.063 | 6.3 | 1.0 | 9.3 | 15.6 |  |  |  |
| 6 | *Andropogon bicornis* L. |  | 38 | 0.088 | 8.8 | 1.0 | 9.3 | 18.1 |  |  |  |
| 7 | *Tonina fluviatilis* Aubl. |  | 38 | 0.088 | 8.8 | 1.0 | 9.3 | 18.1 |  |  |  |
| 8 | *Psychotria poeppigiana* Müll. Arg. |  | 24 | 0.056 | 5.6 | 0.8 | 7.0 | 12.6 |  |  |  |
| 9 | *Isolepis cernua.* |  | 27 | 0.063 | 6.3 | 0.5 | 4.7 | 10.9 |  |  |  |
| 10 | *Dalbergia monetaria* L.F. |  | 54 | 0.126 | 12.6 | 1.0 | 9.3 | 21.9 |  |  |  |
| 11 | *Phyllanthus caroliniensis* Walter |  | 8 | 0.019 | 1.9 | 0.3 | 2.3 | 4.2 |  |  |  |
| 12 | *Scleria gaertneri* |  | 10 | 0.023 | 2.3 | 0.5 | 4.7 | 7.0 |  |  |  |
| 13 | *Fuirena robusta* Kunth |  | 33 | 0.077 | 7.7 | 1.0 | 9.3 | 17.0 |  |  |  |
| 14 | *Clidemia sericea* |  | 22 | 0.051 | 5.1 | 0.5 | 4.7 | 9.8 |  |  |  |
|  | Total |  | 430 | 1.000 | 100.0 | 10.8 | 100.0 | 200.0 |  |  |  |

| Study area | r (soil - roots) | *p* value | r (soil - shoots) | *p* value | r (roots - shoots) | *p* value | Correlation measure |
| --- | --- | --- | --- | --- | --- | --- | --- |
| A1 | 0.860 | 6.874e-08 | 0.818 | 9.992e-07 | 0.832 | 4.612e-07 | Spearman |
| A2 | 0.260 | 0.347 | 0.075 | 0.790 | 0.467 | 0.078 | Spearman |
| A3 | 0.503 | 0.066 | -0.006 | 0.982 | 0.586 | 0.027 | Spearman |
| Ll1 | 0.719 | 0.068 | 0.554 | 0.196 | 0.578 | 0.174 | Pearson |
| Ll2 | -0.107 | 0.838 | 0.385 | 0.450 | 0.676 | 0.139 | Pearson |
| Ll3 | 0.234 | 0.349 | 0.019 | 0.938 | 0.279 | 0.261 | Spearman |
| Ll4 | 0.532 | 0.041 | 0.589 | 0.020 | 0.703 | 0.003 | Spearman |

**Table S3. Correlation coefficient in each study zone, between soil and plant tissues, and between plants (roots and shoots)**

*p*: significance of correlation
